# Odyssey of a Misclassified Genomic Variant: Insight from an Incidental Finding Assessment

**DOI:** 10.1177/2329048X231199327

**Published:** 2023-08-30

**Authors:** Omar Zgheib, Andrea Trombetti, André Juillerat, Siv Fokstuen

**Affiliations:** 1 Division of Genetic Medicine, Department of Diagnostics, 27230 Geneva University Hospitals, Geneva, Switzerland; 2 Division of Bone Diseases, Department of Medicine, Geneva University Hospitals and Faculty of Medicine, Geneva, Switzerland; 3 Division of Internal Medicine, Department of Medicine, 27230 Geneva University Hospitals, Geneva, Switzerland

**Keywords:** epilepsy, genetics, incidental finding, next-generation sequencing

## Abstract

Genetic evaluation of a teenager with seizure found no pathogenic variant in a large gene panel, but an incidental likely pathogenic *HNF4A* variant, deemed to cause MODY1 diabetes. Diabetes history was absent and glycated hemoglobin normal, but serum calcium was severely low, with abnormally high parathyroid hormone. Thus, pseudohypoparathyroidism was suspected and confirmed by molecular genetic testing. Calcium and calcitriol supplementation led to calcium normalization and neurological symptom improvement. Given the absence of personal or family diabetes history, the *HNF4A* variant was reassessed and found to encode an alternative transcript with poor expression and activity levels, hence downgraded on expert advice from ‘likely pathogenic’ to ‘likely benign’. Besides illustrating the importance of structured medical workup before launching extensive targeted exome sequencing, this case highlights the need for caution in incidental finding interpretation in patients lacking compatible phenotype or family history, and the value of expert advice in such variant interpretation.

## Introduction

Since the advent of high-throughput sequencing and its integration into clinical practice, new challenges have emerged, both at the technical level in terms of data management, and also for genomic result interpretation and genetic counseling. More precisely, the impact of a genetic test on diagnosis, prognosis, and potential therapy should be contextualized individually for each patient and not be assimilated in a one-size-fits-all approach, especially not in the case of an incidental finding. The latter is defined as a genomic variant of potential medical relevance unrelated to the medical reason for ordering the test.

The American College of Medical Genetics and Genomics (ACMG) has recently updated its recommendations for reporting of secondary findings in exome and genome sequencing.^1^ In what follows is an odyssey of an incidental finding that impels us to wisely choose and interpret genomic testing and results. This is critical to providing proper medical workup and avoiding unnecessary anxiety for patients and their families, and burden on our health system.

## Case Report

A 17-year-old man presented with a generalized motor seizure at age thirteen and was known for several episodes of abnormal wrist movement felt as twitches since age fourteen. Brain magnetic resonance imaging and electroencephalograms were normal. Upon his pediatric neurologist's advice, genetic counseling and testing was initiated. Family history was unremarkable. Targeted exome sequencing of 2’296 genes associated with seizures as well as developmental and movement disorders was subsequently performed at a private genetic institution. No pathogenic variant was found that could explain the neurological symptoms. However, a novel heterozygous nonsense variant was incidentally identified in the hepatocyte nuclear factor-4 alpha *(HNF4A)* gene, known to cause maturity-onset diabetes of the young type 1. The variant (NM_001287183.1:c.28C > T, p.Gln10Ter) was classified as ‘likely pathogenic’ (class 4) by the laboratory, according to the American College of Medical Genetics (ACMG) criteria**.**^2^ The biological father was not available for segregation analysis. The mother did not carry the *HNF4A* variant.

The patient was referred to an endocrinologist and subsequent diabetes workup was initiated and showed normal glycated hemoglobin levels. Serum calcium was also tested, revealing severe hypocalcemia at 1.28 mmol/L (N 2.20-2.52 mmol/L), for which the patient was immediately hospitalized. No prior blood work for calcium levels was available despite the history of seizures.

On admission, pseudohypoparathyroidism (PHP) was suspected due to elevated parathyroid hormone levels at 72 pmol/L (N 1.10-6.80 pmol/L) in the presence of severe hypocalcemia and elevated phosphorus levels at 1.85 mmol/L (N 0.80-1.45 mmol/L). Thyroid stimulated hormone was moderately elevated at 5.13 mIU/L (0.27-4.20 mIU/L), a finding consistent with PHP. The clinical aspect was unremarkable with no signs of Albright hereditary osteodystrophy phenotype.

PHP represents a wide spectrum of clinical presentations encompassing end-organ resistance to endocrine hormones with or without the Albright hereditary osteodystrophy phenotype.^3^ It is caused either by heterozygous inactivating mutations of the guanine nucleotide binding protein alpha stimulating *(GNAS)* gene on chromosome 20, which codes for the G-protein stimulatory alpha subunit (Gs-α), or by alteration of tissue-specific *GNAS* imprinting*.* The *GNAS* gene had been analyzed within the targeted exome sequencing approach by the private genetic laboratory and did not show any variant. We performed multiplex ligation-dependent probe amplification (MLPA) of the *GNAS* locus in search for a deletion, duplication or an imprinting defect. MLPA revealed an abnormal methylation pattern consistent with sporadic PHP type 1b or inactivating PTH/PTHrP signaling disorder type 3 (hypermethylation of the NESP55 exon and hypomethylation of the AS, XL and A/B exons).^4^ We tested the patient's and mother's DNA samples and found no evidence for paternal uniparental disomy of chromosome 20.

The patient received calcium and active vitamin D supplementation, the mainstream treatment for PHP 1b, which normalized his calcium levels and resolved his neurological symptoms, namely the abnormal wrist movements. The onset of symptoms with the inaugural seizure at age 13 is compatible with PHP 1b. Equally consistent with PHP were enamel hypoplasia and flattening of the growth curve at age 10, with an actual height of 183 cm exactly matching expected height (183 cm), based on his father's (197 cm) and mother's (170 cm) heights.

## Discussion

Owing to the absence of personal and family history of diabetes in this 17-year-old man and the fact that the mean onset age of MODY 1 is 13.8 years, we questioned the pathogenicity of the reported *HNF4A* variant and found that it codes for a transcript that includes an alternative exon (exon 1E), leading to three specific isoforms HNF-4α10/α11/α12. Messenger RNA levels of these HNF-4α-1E specific transcripts have been reported to be 7.2% and 4.2% of total HNF-4α expression in human fetal liver and human adult pancreatic islets, respectively.^5^ A recent extensive study analyzing gene transactivation, expression, and associated transcriptome of each HNF-4α isoform showed that α10, α11 and α12 isoforms had much lower transactivation capacity than α1 and α2, and were not detected in any of the digestive tract tissues examined.^6^ No other study was found on the exon 1E-specific transcripts, likely owing to their low expression levels. These transcripts would arise from a distant upstream *HNF4A* alternative promoter (P2) that drives the expression of other canonical HNF-4α isoforms (α7/α8/α9). The P2 promoter was identified as the major transcription site in pancreatic beta-cells, fetal liver, and liver/colon cancer, while the downstream P1 promoter products are predominant in adult liver.^7,8^

In light of the significantly reduced α10-12 isoform expression levels and activity, we sought advice from experts on monogenic diabetes at the University of Exeter and the Genomics Laboratory of the Royal Devon and Exeter Hospital.^8^ These experts considered the variant to be ‘likely benign’ given its low expression levels.

At this point, it should be recalled that the *HNF4A* variant was an incidental finding in the context of a broad and costly targeted exome sequencing analysis that could have been avoided if serum calcium had been measured in the first place. Clinical PHP would have been identified much earlier and confirmed by MLPA assay for methylation changes, subsequent to normal *GNAS* sequence analysis.

In conclusion, this case report is an anecdotal account of how an incidental genomic finding fortuitously triggered a series of investigations that ultimately led to the correct diagnosis that the extensive exome sequencing intended, but failed, to identify. It highlights the importance of structured and targeted medical workup prior to launching extensive and costly exome sequencing. It also illustrates the need for utmost caution in the interpretation of incidental findings in patients with no compatible phenotype or family history, and the value of expert advice in the interpretation of such variants.
